# Understanding how users of home-based aged care services with cognitive impairment rate their social care related quality of life

**DOI:** 10.1186/s12877-024-05613-x

**Published:** 2025-01-04

**Authors:** Lyn Phillipson, James Caiels, Louisa Smith, Ann-Marie Towers

**Affiliations:** 1 School of Social Sciences, Faculty of Arts, Social Sciences and Humanities, University of Wollongong, Wollongong, Australia NSW Building 29, Northfields Ave, 2522; 2 Australian Centre for Health Engagement, Evidence and Values, University of Wollongong, Wollongong, Australia; 3 Personal Social Services Research Unit, University of Kent, Canterbury, England; 4 Personal Social Services Research Unit (PSSRU), School for Social Policy, Sociology and Social Research, Cornwallis Central, University of Kent, Canterbury, CT2 7NF England; 5 Faculty of Health/School of Health and Social Development/Institute for Health Transformation, Deakin University, Geelong Waterfront Campus, 1 Gheringhap Street Geelong, Vic, Australia; 6 Deputy Director of the Health and Social Care Workforce Research Unit (HSCWRU), The Policy Institute, King’s College London, 22 Kings Way, London, WC2B 6LE England

**Keywords:** Quality of life, Dementia, Cognitive impairment, Social care, Mixed methods, ASCOT

## Abstract

**Background:**

Over the past decades, self-directed models of care have been implemented throughout the world to support older people, including those with dementia, to live at home. However, there is limited information about how self-directed home care is experienced by older people with cognitive impairment and dementia, and how their thinking informs their care choices and quality of life.

**Methods:**

We used the ASCOT-Easy Read, a staggered reveal method, talk aloud techniques, probing questions, and physical assistance to support users of self-directed home care in Australia with cognitive impairment and dementia to discuss their Social Care Related Quality of Life (SCRQoL). Interviews were recorded, transcribed and analysed thematically in NVivo. Demographic, functional, cognitive and SCRQoL scores were analysed in Excel and SPSS. Analysis of both the quantitative and qualitative data for each participant allowed us to examine consistency or discordance between ratings and participants’ comments about their experiences within each domain.

**Results:**

Twenty six older people with cognitive impairment and/or dementia completed an interview. Ratings of SCRQoL were more favourable in lower order domains (e.g. food and drink, personal cleanliness, accommodation comfort and cleanliness and safety) than in the higher order domains (e.g. occupation and social participation). Overall SCRQOL also varied significantly from 0.40 to 0.97. Despite variable ratings, all participants described unmet needs associated with limitations in personal function and mobility, transport and the amount and flexibility of home care services they received. Qualitative comments suggest many experienced more significant limitations than some of their ratings may imply. This was attributed to adaptation and acceptance of limitations as a normal part of aging. The choice to remain living in one’s own home was perceived as the most important outcome.

**Conclusions:**

Some older people living at home with cognitive impairment and/or dementia adapt and accept their limitations as a normal part of the aging process. This affects expectations about their lives at home and their support. Rather than relying on self-direction, supports to live well at home could be enhanced by a greater emphasis on comprehensive needs assessment and more supports to promote reablement and enhance personal and community level participation.

## Background

Globally, there is a strong emphasis on the delivery of home-based care to enable older people, including those living with dementia, to remain in their own homes [1, 2]. Over the past 20 years self-directed models which provide home care users with individualised care budgets were adopted in the UK, US, and parts of Europe [3–6] as well as in Australia [7]. Some studies in the US [8], some parts of Europe [6] and in Australia [9], have shown some improved user satisfaction with self-directed care. However, evaluation in the UK [10] and other Australian research [11] have highlighted that self-direction is more likely to support better outcomes in lower order domains (e.g. having ‘adequate food and drink for health’ or ‘keeping accommodation clean and comfortable’) than it is in supporting high order quality of life outcomes such as ‘social participation’ or ‘choice and control’.

Research specifically with older recipients of self-directed home care in the UK have reported lower psychological well-being for older service users because of the additional burdens associated with planning and managing home care supports [12]. Some older Australian’s living at home, including those with dementia, have also found self-directed care a source of anxiety and confusion [13, 14] due to their limited understanding of the supports and options available to help them live well at home [15]. In Australia, a survey of home care providers found that the vast majority (86.2%) believed people with dementia with no active carer/advocate were less well suited to self-directed care [16]. Others have also reported that because older people with cognitive impairment experience more difficulty with self-direction, they spend more of their care budgets on case management and less on direct services [17].

Community-dwelling people with dementia who still live at home reportedly value: access to social contact and company, feeling safe and secure, feeling financially secure, being personally clean and comfortable, living in a clean and comfortable environment and a degree of autonomy and control as core values [18]. Safely staying at home with personalised activities is also valued by people with dementia and their carers [19]. These domains of quality of life, plus additional outcomes concerning maintaining personal identity, communication and managing dementia symptoms were confirmed through a Delphi process with people living with dementia in the community in the UK [20]. However, the extent to which any of these outcomes are experienced by people with dementia who are living at home and receiving self-directed home care, is currently unknown.

In this study we aimed to understand more about how older recipients of self-directed home care with cognitive impairment and dementia evaluate their care-related quality of life and the extent to which it helps them to live well at home.

## Methods

We used an adapted Easy Read version of the Adult Social Care Outcomes Toolkit (ASCOT-ER) in interviews using a staggered reveal method and other assistance where needed (e.g. hearing, visual or physical assistance with writing) to support assessment of self-reported social care related quality of life (SCRQoL) [21, 22]. The eight domains of the ASCOT cover core or lower order aspects of SCRQoL including personal cleanliness and comfort, accommodation cleanliness and comfort, food and drink, feeling safe and also higher order aspects including social participation, occupation (how you spend your time) and control over daily life. The eighth domain, dignity, covers the impact of care on how people feel about the people who provide them with care [23].

The ASCOT-ER format uses black and white illustrations and plain text to convey the meaning of each quality-of-life domain. The selection of response options is supported by a visual scale and text-based response categories. The cognitive interviewing protocol included a staggered reveal method, talk aloud techniques and probing questions to explore understanding of the pictures, questions and why particular answers were selected [23–25]. Participants in this study were engaged in a discussion about each domain and then asked to rate their outcomes. Two researchers, both with clinical experience of people with dementia, collected the data, in person, in the homes of participants. In 1/3 cases, the care partner was also present during the conduct of the interview at the request of the participant.

**Fig. 1:**
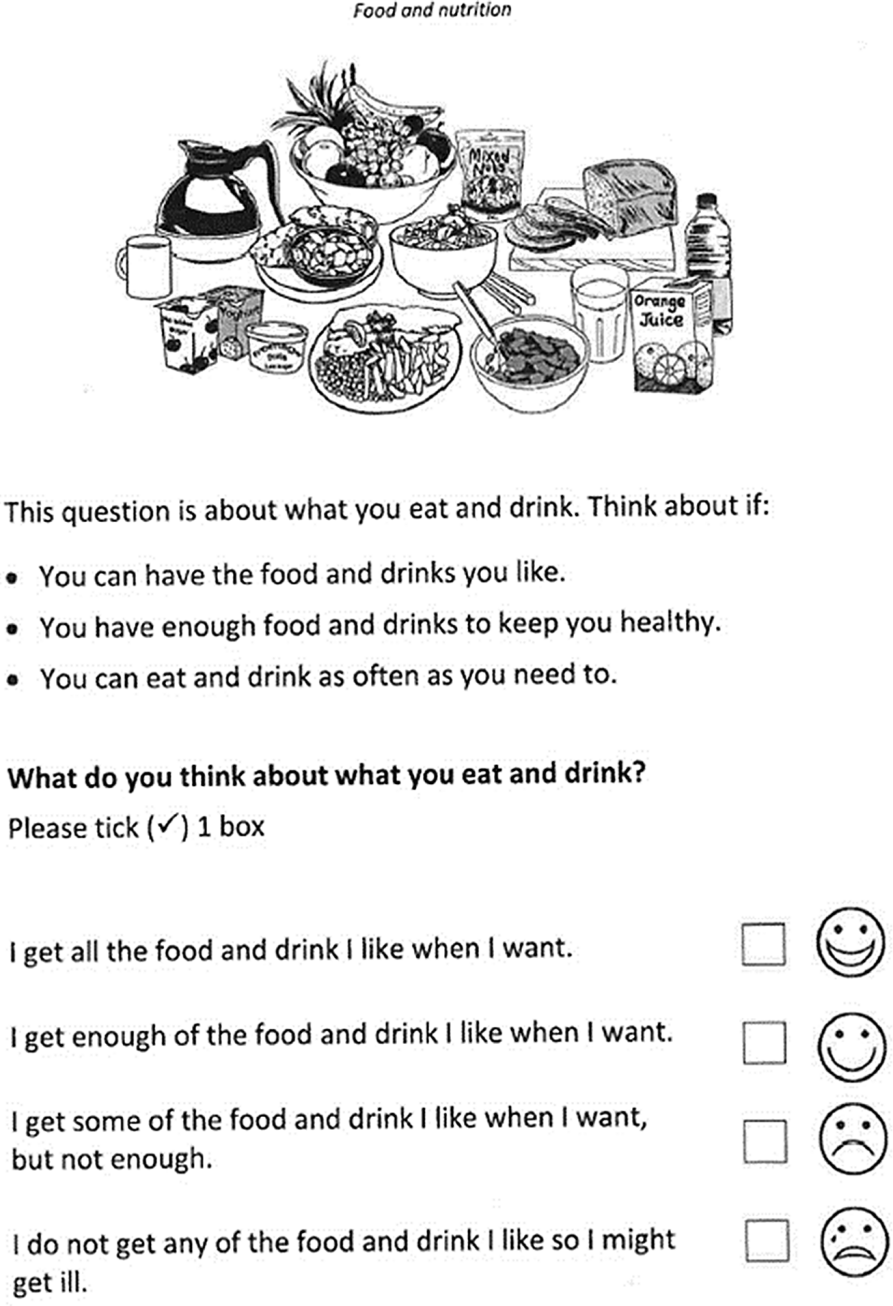
Sample of ASCOT-ER format: Food and Drink Domain © University of Kent. Reproduced with permission. All rights reserved

The ASCOT-ER was chosen for: its accessible format; the similarity between the domains assessed and those identified as important by people with dementia [18, 20] and consistency with areas in which the Australian home care program provides support [24]. The explanatory text and some images were adjusted slightly during the study to enhance its appropriateness for the older cohort with cognitive impairment. This resulted in some of the sample using the original ASCOT-ER and others using a slightly modified version. However, analysis of these two cohorts showed there were no significant differences in their demographic characteristics or the way they rated their QoL [22]. See Fig. 1 for an example of the ER format question stem and pictures for the Food and Drink domain.

### Recruitment and characteristics of participants

Inclusion criteria required participants to be living within the community and receiving aged care services through the Home Care Packages (HCP) Program [24]. The HCPs assist individuals aged 65 and older who require coordinated care and services to remain living at home. The program operates under a consumer-directed care model giving individuals the flexibility to choose their provider and services within the scope of an allocated annual care budget. This budget ranges from Level 1, addressing basic care needs (around AUS$10, 588.65), to Level 4, which covers more complex care requirements (up to AUS$61, 440. 45) [26]. Available services may include help with personal care (e.g., bathing, dressing), nutrition and meal preparation, continence care, support with mobility aids, and, for high-level packages, access to nursing, allied health, or other clinical services. To be included, all participants were also required to have suspected or confirmed cognitive impairment or dementia.

Both providers were recruited through invitations emailed by the lead researcher to all HCP providers in the Illawarra and Southern Highlands region. The invitation was to participate in the testing of the ASCOT-ER tool with users of home care packages with cognitive impairment [22]. Participant recruitment was done via the two service providers who agreed to be involved. This was essential to ensure that participants received suitable help with both recruitment and with follow-up support required after their involvement in the research. Service providers gave participants written information before the interview and were asked if they were interested in participating in the research. When this occurred, researchers then made contact with potential participants to support them to review the details in the written information, answer any questions, and to clarify their understanding before gaining their written consent. If the researchers assessed the participant as unable to provide consent, then proxy consent was sort via a carer or guardian. Consenting participants were then monitored throughout the research process, to confirm their ongoing willingness and interest in participating [26]. The University’s Human Research Ethics Committee granted approval for the study (HREC Approval 16/236).

### Data items

Demographic data, was collected face to face by two researchers, included: age, language spoken other than English (LOTE), gender, carer status, carer co-residence, education level, self-reported diagnosis of dementia, perceptions of monthly family finances (not enough to make ends meet; just enough to make ends meet; some left over) and the level of home care package support (Level 1–4). Cognitive status was screened using the Mini-Cog© (a score of < 3 was used as an indication of cognitive impairment) [27, 28]. General functional ability was assessed using the National Home and Community Care (HACC) functional screening test which includes questions about instrumental IADLS (e.g. ability to shop, do groceries, prepare meals) and ADLs (e.g. walk, take a bath or shower). The HACC screener has a maximum score of 16, with a lower score indicating more difficulty managing with daily activities of living [29, 30].

### Analysis

Whilst all 26 participants were able to discuss each domain within the interview, only 24/26 were able to rate their outcomes within each of the 8 domains ASCOT SCRQoL scores. Scores for those 24/26 participants were entered into Excel (1 = ideal state, 2 = no need, 3 = some needs, 4 = high needs). Overall SCRQoL were derived from weighted population preferences of different aspects reflected in the domains (not bespoke to Easy read). These were summed to give a total ranging from − 0.17 to1.00, with 0 equating to ‘being dead’ and 1.00 to ‘ideal’ state, below zero represents a score of being worse than death [23]. The ASCOT-ER differs from other tools in the suite e.g. the ‘Safety’ domain asks two separate questions to distinguish between safety in the home and that experienced in the local area. This reflected testing with adults with intellectual and developmental disabilities who wanted to express differences in how they felt about their safety in different settings [21] and is not something that had arisen previously when the main measure was developed and tested with older people [23, 31]. To enable the calculation of an overall SCRQoL score using the weighted preferences, and comparability with the other ASCOT measures, we followed the recommended guidance and used the safety score indicating the highest need for each participant [21].

Data were analysed using SPSS 24 [32]. Categorical data were examined using Chi-squared or Fisher’s exact test and continuous variables were examined using independent *t*-test (*p* < 0.05) to compare the demographic characteristics and ASCOT domain scores between the original and modified versions of the ASCOT-ER used in the study. Levene’s test was conducted for t-test to homogeneity of variance. Comparisons of participant responses between the two versions of the survey (original vs. modified) showed no difference in either the demographic characteristics or their ratings of their quality of life suggesting that modification of the tool had not altered its validity. Results were therefore analysed for the cohort as a whole, including descriptive analysis (response counts and percentages) for each ASCOT-ER domain.

### Qualitative

Cognitive interviews were audio-recorded and transcribed verbatim for analysis in NVivo 11. All 26 transcripts were analysed by Author 1, Author 2 and a trained research assistant. Initial inductive coding was discussed and refined over 2 or 3 meetings to gain agreement on overarching themes. These included a common set of personal and service-related factors that affected experiences within each SCRQOL domain (see Table 1).

**Table 1:** Inductive codes and agreed themes of factors influencing evaluation of outcomes for each SCRQOL domain

Domains	Examples of Inductive coding	Overarching themes
**Accommodation cleanliness and comfort**	Pain, can’t walk, bend over, stand, vacuum, hang out clothes. sight	Mobility/Function
	Can’t afford more cleaning, nicer furnishings etc., can’t afford to heat or cool	Finances
	No one ever visits, no need to keep clean for anyone else	Social aspects
	House proud, don’t care, temperature, soft furnishings	Preferences
	Motivation	Emotional
	Don’t come frequently enough to meet needs	Service Factors
**Personal Appearance & comfort**	Pain, Walk, drive to the hairdresser, sight, can’t shop so can’t choose clothes	Mobility/Function
	Can’t afford the clothes Ilike to wear, can’t get to the hairdresser	Finances
	No one sees men nowhere to go out too	Social aspects
	Soft, warm, comfortable	Comfort
	Wanting to get dressed up, feel special, have hair done, don’t care	Preferences
	Motivation,lLoss of self-esteem, don’t feel good about self	Emotional
	Don’t come frequently enough to meet needs	Service Factors
**Food and Drink**	Walking, standing, transport to shops or restaurants, taste. Sight, smell, can’t get to the shops so can’t choose food, can’t walk around the shops, can’t cook so can’t eat what I like	Mobility/Function
	Not enough money to buy enough food or the food I like	Finances
	Eating with other, cafes, restaurants, special occasions	Social aspects
	Health, taste, plain, light, can’t eat what I like, favourite foods, cultural, comfort	Preferences
	Motivation, just me, can’t be bothered, cant taste anything	Emotional
	Don’t have time to take me shopping, don’t buy the things I prefer	Service Factors
**Occupation**	Walking, transport, need assistance to do things, can’t see, can’t hear, can’t dance	Mobility/Function
	Can’t afford to do things they enjoy, or transport to do those things	Finances
	No one to share activities with	Social aspects
	Opportunities, nothing to do	Preferences
	Motivation, apathy, lost interest, no enjoyment anymore, loss	Emotional
**Social**	Hearing, sight	Mobility/Function
	Limited money to go out and socialise	Finances
	No one to do anything with	Social aspects
	Don’t need it, really like it, prefer family, only want to see my old friends, happy to talk to anyone, always been anti-social, pets, limited opportunities	Preferences
	Grief, loss, loneliness, limited access to friends, partners, family members, motivation, lost interest in social contact	Emotional
	Good relationships with some carers, don’t always get the carers they prefer	Service Factors
**Safety at home**	Falls, can’t defend self	Mobility/Function
	Neighbours, living alone, carer	Social aspects
	Personal alarms, walking aides, railings, Workers checking in, faith, pets, leaving lights on, TV on, making it look like they are not alone	Preferences
	Fear of being alone, worry about falling over, injury, being robbed	Emotional
	Workers checking in	Service Factors
**Safety Neighbourhood**	Falls, walking in crowds, uneven footpaths	Mobility/Function
	Neighbours, local people dealing drugs, drunk, erratic behaviours, youth especially feared	Social aspects
	Stay inside, go out with companion, Won’t go out at night, familiar neighbourhood	Preferences
	Feels threatened, fear, comfort	Emotional
**Dignity**	As people need more help they feel more grateful	Mobility/Function
	Staff cant visit enough due to low package	Finances
	Staff, kindness, being listened too, nothing too much trouble, staff are friend or family	Social aspects
	Same staff, familiarity with staff, predictable times and days, reliable	Preferences
	I’m not a bother, happiness, feel happy,	Emotional
**Control**	Can’t walk or get around in the community, can’t drive	Mobility/Function
	Not enough money to buy what is wanted or needed	Finances
	Ability to choose carers they like	Social aspects
	Ability to choose	Preferences
	Motivation, don’t want to be told what to do, want autonomy	Emotional

### Mixed methods analysis

We ran coding queries in NVivo that enabled us to analyse and compare the transcripts for each domain ‘grouped’ according to their rating of that domain. This enabled qualitative comparison of factors associated with reports of an ‘ideal’ or ‘no needs’ state compared with a rating of ‘some needs’ or ‘high needs’ within each domain [31]. Results from the mixed methods analysis are presented to give context to the participants’ ratings, highlighting comments which describe both their lived experiences and informed their self-rating. Following thematic analysis, we were able to identify participant quotes and comments and associate these with each participants’ ratings. This enabled us to examine consistency or discordance with each rating provided, and the comments provided by participants about their experiences within each domain.

## Results

Table 2 (below) highlights the characteristics of the *n* = 26 participants who took part in the study. Their ages ranged from 63 to 99 years (M = 82.51). Two spoke a language other than English (LOTE) (2, 7.7%). Just over half were female (15, 58%), 71% reported having a carer, though less than one quarter of these were co-resident (6, 23%). All participants were screened as having cognitive impairment (scored less than 3 on the Mini-Cog). However, only just over a third reported they had a confirmed diagnosis of dementia (9, 35%). The mean HACC function score was 9.5 (3.07), indicating participants, on average, were in need of some help with a majority of their IADLs. Participants were supported with different levels of home care packages including: lower level 2 care packages (16, 62%) which provide a budget to meet basic care needs, whilst one third received higher budgets to meet Level 3 (intermediate) or Level 4 (high care needs) packages (8, 31%) and two were unsure of their level of support.

**Table 2:** Participant characteristics

Demographics	Total, *n* = 26
Age (Years), Age, Mean (SD)	63–99 82.51 (9.89)
LOTE, yes *n* (%)	2 (7.7)
Gender, Female *n* (%)	15 (57.7)
Education, high school or more, *n* (%)	20 (76.9)
Finances, some leftover, n (%)	17 (65.4)
Care partner, yes, *n* (%)	19 (73.1)
Co-resident care partner, yes, *n* (%)	6 (23.1)
Diagnosed Dementia, yes, *n* (%)	9 (34.6)
Mini-cog, mean (SD)	2.31 (1.62)
HACC, mean (SD)	9.50 (3.07)
Home Care Packages, Level 2, 3 or 4 (n, %),	Level 2 (16, 62%) Level 3 (1, 4%) Level 4 (7, 27%) Unknown (2, < 1%)

### Current social care related quality of life

Table 3 (next page) describes how current home care users with cognitive impairment and/or dementia rated their current SCRQoL. Overall average SCRQoL was 0.74 (noting that a maximum score of 1.00 indicates participants feel they are in an ‘ideal state’). However, a clear pattern emerges when examining how SCRQoL varies across the different domains, especially when comparing lower order and higher domains. For example, in the lower order domains, those that are essential to life (personal cleanliness, food and drink, personal safety, accommodation), we found the proportion of participants rating themselves as either in an ‘ideal’ or ‘no needs’ state to be between 87.5 and 95.9 per cent. These ratings indicate that most people rated their needs and preferences in these areas as being met. When examining the higher-order domains (control over daily life, social interaction, occupation), the prevalence of no needs or ideal state ratings was noticeably lower, ranging from around 43.5 to 75 per cent, indicating that SCRQoL is lower in these domains. Analysis of qualitative comments revealed common themes across all domains as affecting experiences in each SCRQOL domain. These factors included both personal factors (e.g. related to mobility/function, limited finances, unmet preferences, emotional factors) and service factors (e.g. limitations in services provided, willingness and ability to meet preferences) all contributed to unmet need.

Importantly, mixed methods analysis also revealed that many participants who rated themselves as in an ‘ideal’ or ‘no needs’ state were actually experiencing more limitations than their ratings suggested.

**Table 3:** Social care-related quality of life (SCRQoL) ratings by Adult Social Care Outcomes Toolkit (ASCOT-ER) domain

ASCOT-ER SCRQoL Domains	Ideal State	No needs	Some Needs	High needs
**Lower order domains (core)**				
Personal cleanliness and comfort, n (%)	7 (29)	14 (58)	2 (8)	1 (4)
Safety (Home), n (%)	10 (42)	12 (50)	1 (4)	1 (4)
Safety (Local Area), n (%)	10 (42)	12 (50)	2 (8)	0
Accommodation cleanliness and comfort, n (%)	11 (46)	12 (50)	1 (4)	0
Food and Drink, n (%)	13 (54)	10 (42)	1 (4)	0
**Higher order domains**				
Occupation, n (%)	4 (17)	6 (26)	11 (48)	2 (9)
Social participation, n (%)	7 (29)	6 (25)	10 (42)	1 (4)
Choice & Control, n (%)	9 (38)	9 (38)	6 (25)	0
**Care processes**				
Dignity, n (%)	21 (91)	2 (9)	0	0
**Overall**,** SCRQoL**	0.74*			

*Calculated using highest needs score from the safety domain in either home or local area

### Lower order care domains

#### Accommodation cleanliness and comfort

Most respondents rated their homes as in an ideal state ‘*My home is as clean and comfortable as I want*’ (*n* = 11, 46%) or as having no needs ‘*My home is quite clean and comfortable*’ (*n* = 12, 50%). Only one rated themselves as having some needs. This related to her ability to have the kitchen clean prior to her participation in the research interview. Her comments suggest both a limitation in the amount of services received and also a lack of flexibility to respond to her needs, in this case, making her home more presentable for a visitor. This was described as having an emotional impact.

**P6:** ‘My kitchen and my fridge are really bad at the moment. I was thinking about the kitchen mainly. I’m quite happy with [the other rooms] like this. It’s a bit messy but the kitchen’s absolutely disgusting… I was trying to clean up before, hoping I got there before you came…but my back is bad and I slept all night on the couch…[and they can’t come enough to help me keep on top of it]….it’s a bit embarrassing really.’[Some needs].

#### Personal cleanliness and comfort

Respondents most frequently rated their personal cleanliness and comfort as being in a ‘no needs’ state ‘*I feel quite presentable. It is ok*’ *(n* = 14, 58%), with the next most frequent response an ideal state, ‘*I feel very presentable*’ (*n* = 7, 29%). When discussing their responses participants mostly reflected on the importance of feeling clean, warm and comfortable.

**P7:** ‘Look, to be presentable, cleanliness first of all I’d say.’ [Ideal state].

**P19:** ‘Well I just feel comfortable that’s all**….**I just dress for comfort not for, not for, not for good looks …or anything like that, no.’ [No needs].

Only two people (*n* = 2, 8%) rated some needs and one (*n* = 1, 4%) high needs in this domain. They described financial limitations restricting their ability to buy comfortable clothes and one also mentioned not being able to have her hair done the way she preferred. The inability to present herself comfortably had a negative impact on her sense of self.

**P6:** ‘Well, I’m just thinking I can choose to go up to [the supermarket] if I want to … but I’d like more choice because I’d like to have more money and I’d love to go and buy some clothes [elsewhere]. I mean okay, when I go out I don’t want to look like this…I’ve got no clothes that fit me. My hair needs cutting and colouring. I haven’t got that done…’ [High needs].

A number of respondents also reported limitations regarding their personal cleanliness. This included people who rated their cleanliness as ‘ideal’ or ‘no needs’. The common experience for each was not being supported to shower or wash clothes as frequently as they would like, indicating a limit to the amount of services they received.

**P4:** ‘[I am] helped by having an assistant … but I can’t shower as often as I might like.’ [No needs].

**P11:** ‘No my clothes are scruffy. I haven’t washed for a week and that’s as often as it gets done.’ [Some needs].

**P3:** I feel presentable, yeah. Well at least most of the time. But if I am being honest, not all the time [Ideal state].

Whilst many were happy with dressing for comfort, others described a lack of motivation to attend to their appearance unless they were expecting social contact. Some were not able to adequately present themselves for these interactions which resulted in negative feelings about themselves.

**P11** ‘I feel presentable when I go out on a Wednesday, that’s the only day I ever bother.’ [Some needs].

**P6:** ‘I don’t feel like putting an effort in, plus it’s painful. Even getting dressed is painful. So, my appearance is just dreadful.’ [High needs].

**P26:** **‘**Well when I meet a friend who’s immaculate and hair [is] always done and she’s slim, and dresses nicely I always feel like I’m a bit of a bag.’[Some needs].

#### Food and drink

The vast majority rated their access to food and drink as ideal, ‘*I get all the food and drink I like when I want*’ *(n* = 13, 54%) or having no needs, ‘ *I get enough of the food and drink I like*’ (*n* = 10, 42%). When discussing their response options participants mentioned the ability to do their own shopping as important.

**P4:** ‘I go do the shopping myself. I buy everything I like, all the drinks and food.’[Ideal state].

**P4:** ‘I *make it* [emphasised] that [the support worker] put in my trolley what I like.’ [laughs] [Ideal state].

Only one respondent rated themselves as having some needs in this domain, ‘I get some of the food and drink I like when I want’. She was dependant on others to shop for her, and this limited her control and choice. Her comments indicate some limitations in the amount and flexibility of the services to meet her needs. Another participant rated himself as ‘no needs’ but suggested the limited frequency of his shopping restricted his access to fresh food and his ability to offer food to visitors.

**P5:** ‘I get some of the food and drink I like when I want it, but not enough….I think I would have to say that there… because like I say it [the fresh fruit] runs out, because I’d like to get more’ [Some needs].

**P23:** ‘Well as long as I remember to order enough on Wednesday so that I can go ‘til the following Wednesday. And of course, if I eat one extra piece of fruit or something or other, well you’re without it. Or if anybody comes and eats your fruit or – you’ve got to ration it based on when you can get it again…’ [No needs].

Despite rating themselves as ‘no needs’ other participants revealed their food preferences were not met. Not being able to cook for themselves limited some respondents’ choice and access to food they preferred.

**P13:** ‘[My husband] does…all the cooking [and] sometimes we get some things cooked for us [by the service provider]. [I’d like more] Baked dinners. A roast. We’re [also] down on the veggies a bit…’.

**Husband:** ‘Oh yeah, she wants things that I don’t like, so I don’t bother cooking it.’ [No needs].

Some participants missed being able to have food they associated with special occasions or going out for a meal.

**P6:** *‘*I get all the food and drink I like when I want really, although it would be nice to go to a restaurant occasionally…It’d be nice to have fancy food occasionally and I don’t do that anymore.’ [No needs].

### Safety in the home

The most frequent response in this domain was to indicate no needs ‘*I feel quite safe in my home’* (*n* = 12, 50%) or an ideal state ‘*I feel very safe in my home’* (*n* = 10, 41.7%). Equipment and technology were important in providing a sense of safety. This included use of personal alarm systems and walking aides. The familiarity of home, having a personal faith and pets were also mentioned as contributing to a sense of safety.

**P6:** ‘I’ve got this thing [personal alarm] around my neck. Yeah, it’s like I know if someone broke into my house, I could just push this button and someone can hear me.’ [No needs].

**P7:** ‘I rented this [walker] from the hospital. Without this I would have many falls.’ [No needs].

For the two, who *‘do not feel safe enough in my home’* (*n* = 1, 4.2%) or ‘*do not feel safe in their home at all*’ (*n* = 1, 4.2%) the perception of living in an unsafe neighbourhood specifically, and also in an unsafe world more generally, interfered with their sense of personal safety when inside their homes.

**P5:** ‘I don’t feel real safe, because I have been attacked here…and I’m always wondering, is it going to happen again… that’s how I see it…and then you’re frightened… all the time.’ [High needs].

**P24:** ‘Well that’s a very hard question…the way things are going in this world. People are getting killed in their home or they’re getting bashed up…’ [Some needs].

One participant also mentioned a fear of being bullied or intimidated in her home in response to the Easy Read picture in the questionnaire. However, she rated this domain as ‘no needs’.

**P6:** ‘She’s getting bullied [in that picture]. My son talks like that to me sometimes….it’s not nice.’ [No needs].

### Safety in the local area

The most frequent response in this domain was to indicate no needs ‘*I feel quite safe in my local area*’ (*n* = 12, 50%) or an ideal state ‘*I feel very safe in my local area*’ (*n* = 10, 42%). When discussing their response options, safety when out and about in the local area was supported by familiarity with the local area. Others achieved safety by adapting to their disability or restricting their activities. For example, walking to familiar places, only going out in the daytime or with an escort or not going out at all.

**P3:** ‘No, I feel very safe here. As I said before, I walk a lot… I know the area so well. I don’t have any fear of getting lost that I would have with [my] dementia maybe somewhere else, yeah… But I would only go in the day…But yeah, [at night] I think about self-protection, I guess. You’re old enough to know things can go wrong.’ [Ideal state].

**P4:** ‘…well actually if I’m in company [Yes]. When I’m alone I’m worried.’ [Ideal state].

**P5:** ‘Well you’ve got drunks….There’s a woman over there sells drugs. And you’ve got a police car coming up here at least twice in 24 hours and maybe more. And I’ve been checking them, and they’ve got a lot of drug mules… I’m not safe, so I don’t go out.’ [No needs].

Despite many expressing concerns about safety in their local area, only two participants (*n* = 2, 8%) rated themselves as having some needs.

### Higher order domains

With regards to the higher order care domains, Control Over Daily Life (71%) was rated as the domain of lowest need. However, Occupation (having meaningful activities to do) (51%) and social participation (seeing people they like as often as they like) (60%) were the domains of highest need. In some of the higher domains, qualitative comments revealed significant limitations experienced by those who rated themselves in an ‘ideal’ or ‘no needs’ state.

### Social participation

Over half the cohort rated themselves as having some needs ‘*I see the people I like but not enough. It could be better*’ (*n* = 10, 42%) or high needs, ‘*I do not see the people I like at all. And I feel lonely’* (n = 1, 4%). Respondents talked about loss, limitation, loneliness and longing. Some described how important relationships had ended, and many reflected on the past when they felt less lonely and more connected.

**P19:** ‘Well - well we don’t socialise as much as we use to because [clears throat], um, most - most of our old neighbours are dead…. and our social life has diminished because of my - my condition and [my wife] can’t walk … Every second week we use to have six or eight of our friends over ….and we’d have a dinner first and then we play cards… we don’t do that now…’ [Some needs].

**P8:** ‘Everyone I know has either died or shifted out and there’s new people come in here. You come up the main street now and you know nobody.’ [Missing rating].

**P6:** ‘How do I feel about [my] social life? It’s zilch. No, it’s happened gradually over the years. I’ve just become more and more isolated … I feel lonely.’[Some needs].

**P4:** ‘Sometimes it is sad. Nobody comes…I miss my husband … and then I sit down and cry [laughs]. But I reckon everybody does that, because we always have our mourning.’ [Some needs].

For those who rated themselves as having ‘no needs’ i.e. ‘*I see people I like enough’*, the proximity of family was an important buffer and support.

**P9:** ‘Oh, I see all the people I like as much as I want to, and my family is around. I had a great time, on the weekend all the grandchildren came around. My two daughters live nearby. My son comes up and stays with me a few days a week from Canberra.’ [Ideal state].

Neighbours took on a new importance due to their proximity. However, this was not always sufficient to replace the longer-term relationships of importance or to negate the need for more contact.

**P4:** *‘*I love my social life. I love to talk to people … [laughs], anybody, everybody. I mean all my neighbours, I really have lovely neighbours, and it’s the same thing …but I mostly would love to see my family and my [old] friends.’ [Some needs].

The regular contact with service workers was an important part of socialisation. Respondents emphasised that a cup of tea and a chat was as important to them as any assistance they received with instrumental activities. Care workers were frequently the only people they had regular contact with, and some described them as ‘like family’.

**P11:** ‘My social life is okay. I never see, I never see anybody, but it doesn’t worry me, ‘cause I see [the worker], I’m quite happy with [the worker], they come down every day at 4 o’clock… I love my girls, I call them my daughters, much to my daughter’s disgust.’ [Ideal state].

Respondents’ limited mobility and access to transport limited their social contact. Not being able to drive was frequently associated with need, along with the inability to walk as far as they wanted to. However, many participants appeared to accept and adapt to their shrinking world as a normal part of aging.

**P2:** *‘*So …it’s a happy life in one sense that I’m still able to do what I want to do [at home] but I can’t drive a car anymore and I can’t get out. I have to wait for somebody to take me out…. But I make the best of it….at my age to be still walking but I can’t get out. So the car’s kaput so who’s going to take me out, love, they’ve got their own families and I do get lonely sometimes.’ [High needs].

**P4:** ‘Yeah, I love socialising….It can be my neighbours or my friends, or just anybody. When I used to be younger, I used to just go out for walks and I sit on a bench, talk with anybody that just comes along sitting down next to me.[But] ‘I’m not that good on my feet… I had a stroke and ever since it gets hard for me to walk sometimes, like I get worn out, I’ve got to sit…. I used to walk from here to my daughter’s place, and back. But I’m not as good anymore…it’s my age.’ [Some needs].

Others described their limited social contact as consistent with their life-long habits of keeping to themselves and resisted support on the basis of long-standing preferences.

**P11:** *‘*[The service provider] tried to get me to go out and socialise when they first came here, did I want to go out to join anything that was on. I said, “no”, ‘cause I’ve never done it and I don’t want to do it. I’m just happy for my own…and that’s me…’ [Ideal state].

### Occupation

With regards to occupation, over half the sample rated themselves as having some needs ‘*I do some of the things I like. But I would like to do more*’ (*n* = 11, 48%) or high needs (*n* = 2, 9%) ‘*I do not do the things I like. It is really bad*’. When choosing their response options, participants with high needs felt very limited in what they could do.

**P2:** ‘I’m afraid to say I go to bed. I take myself in and hope to have a sleep and get through that part of the day.’ [High needs].

Respondents with both ‘high’ and ‘some needs’ attributed these to their loss of function or transport. Many reported needing assistance to do most of their activities, or an inability at all to do activities that used to bring them enjoyment.

**P26: ‘**I don’t know. It’s not what you’d like to do anymore…it’s what you can do.’ [Some needs].

**P2:** *‘*Well…somebody has to be with me all the time to get me out and bring me back and the women [the care workers] don’t want to do that, love. They’ve got their own families and their own husbands to look after so they just dump you but then they’re very nice about it.’ [High needs].

**P23:** *‘*Well I’ve got lots of good free time, but can’t use it as I want to… Well again we’re back, thrown back to transport…one of my remaining activities is reading. But I’ve still got to be able to get to the library!’ [Some needs].

Not having enough meaningful activities had an emotional impact, creating a sense of frustration and worthlessness. This was evident both in those who rated themselves as having needs, and some that did not.

**P21:** ‘Sometimes I feel useless. Other times, I fall asleep. I sleep a lot.’ [No needs].

**P26:** ‘Frustrated is the word. Yes. You can’t do things that you would like to be able to do.’ [Some needs].

**P4:** ‘I sit here feeling a bit useless. Not enough to do.’ [Some needs].

Respondents also discussed low motivation to engage in activities, frequently due to having no one to share activities with. This highlighted an interaction between responses in the social and occupational domains.

**P2:** *‘*I used to love sewing, I used to love cooking but for me what is it for now? I don’t bother, love, somebody comes and helps me and I’m really appreciative of it, but it wasn’t what I expect old age to be… I used to like doing things but I don’t like doing anything like that now, you know, embroidery, well what do I want to embroider love?’ [High needs].

The other third of the cohort rated themselves as being in an ideal state, ‘*I spend my time how I want. It is great’* (*n* = 4, 17%) or as having no needs, ‘*I do enough of the things I like. It is OK’* (*n =* 6, 26%). However, some participants seemed resigned to their low levels of activity, choosing a ‘no needs’ response despite describing themselves as having nothing to do. This was associated with an expectation of old age as a life stage lacking activity and an acceptance of their limitations such as limited mobility.

**P1:** ‘I do nothing. [Laughs] I look in TV. I do puzzles, yeah. I try to do puzzle, finish. I don’t read… I’m too old to have something to do. I do enough. I’ve got enough.’ [Ideal state].

**P21:** ‘Well yeah, I can’t do things I enjoy….I cannot go to the meetings, like when they have a turn out or things like that, because I can’t get around. That’s where I’m useless. I just accept that I can’t get around at my age.’ [No needs].

### Control over daily life

Despite the limitations described in their interviews, most of the respondents rated their control and choice as in an ideal state ‘*I have as much choice as I want. It is great’* (*n* = 9, 38%) or as having no needs, *‘I have enough choice. It is OK’* (n = 9, 38%).

Choices that informed their response options included: choosing where to shop, what they would like to spend their money on, and choosing what they wanted to cook and eat. Some felt that having choice was important and they felt in control. Others however described doing things because others thought it was good for them.

**P19: ‘**Oh, I think I have a lot [of choice], and it should all, all fall on me, what I should have, what I should do, what I should eat.’ [No needs].

**P18: ‘**Well I, um, I have some freedom to make my own decisions…[but] sometimes I have to, because somebody, not necessarily a wife…you could be the man next door…makes me do something because he thinks it’s good for me or something.’ [No needs].

**P15: ‘**Can I go out? I can do whatever I like…. Other than, other than me sister says I can’t do it, well, I don’t do it….She’s the boss.’ [Ideal state].

Some who rated themselves in an ‘ideal state’ had also adjusted to a life of simple desires and expectations.

**P20:** ‘‘Choices? Well, these days… like we do – we do, watch a bit of television. And we sometimes we sit out in the sun, we like doing that. On a sunny day. But there’s no way of getting anywhere else, so there’s not much to decide.**’**[Ideal state].

Six participants (25%) who rated themselves as having some needs discussed their choices being limited by their mobility. These respondents desired better mobility and transport to assist them to go beyond the home.

**P21:** ‘Choices is I’d like to be able to walk properly. Another thing, I’d like to be able to drive. I had my licence taken off. I just can’t take part in the world anymore. It’s like I don’t exist’ [Some needs].

**P23:** ‘‘Well there are limits to all of these factors, and that limit is transport.’ [Some needs].

**P26:** ‘We’re both restricted because of our mobility.’ [Some needs].

**P3:** ‘Yeah…my choice is good but not excellent, yeah, because I don’t drive anymore and that’s a real, real issue. I can’t get anywhere without sort of getting somebody, and that doesn’t happen very often because both my daughters work full-time.’ [Some needs].

Finances were also seen as limiting people’s choices.

**P6: ‘**Not much choice because of the restrictions of the money.’ [Some needs].

For some, they appeared to have low expectations of life at their age and stage. This at times, made the question difficult question to answer, particularly for those who felt very limited in their functioning.

**P2:** *‘*What is in my daily life? I get up, have my breakfast, if any, if I feel like it, if not I don’t. I go to bed when I want to, sometimes I go to bed in the afternoon ‘cause I’m bored, and I’m fed up. I kick the dog, go out and see what’s outside, take the thing in and that’s it, love. What choices? What choice am I supposed to have [at my age]?’ [Some needs].

Participants identified that the choice to be living at home (rather than in a nursing home) was the most important contributor to a sense of control.

**P2:** ‘Well I’m here, aren’t I? That’s my main choice… No I wouldn’t want to go to a home…I wouldn’t like somebody bossing me all the time, love, not while I’m capable of cleaning up the mess I make…’ [Some needs].

### Dignity

Dignity in Care was the highest ranked domain overall, with the overwhelming majority rating their experience as in an ideal state ‘*I am very happy with the way my paid support treat me*’ *(n* = 21, 91%) with the other two participants indicating no needs, ‘*I am quite happy with the way my paid support treat me*’ (*n* = 2, 9%).

Kindness and respect, as well as relational elements which incorporated caring, friendship and staff who were ‘like family’ were described by respondents when considering their lived experiences and ratings.

**P2:** ‘Whoever comes to see me they’re more than that, they’re very, very kind, love.’ [Ideal state].

**P4:** ‘Yeah, paid support; fantastic, they treat me fantastic. None of them ever had a bad word for me or insulted me in any way or accused me or whatever…I feel very happy, the way I get treated with respect, with friendship.’ [Ideal state].

**P11:** ‘Oh, I think so… I always say, “They’re my family that I can’t have”, yeah, I think the world of them, really. They are, they’re my family, I always treat them like my family, they’re very special to me.’ [Ideal state].

Overall, when choosing their response options, participants were overwhelmingly thankful for their support and saw it as essential to achieving their ultimate goal – to remain living in their own homes.

**P7:** ‘Those girls [the care workers] kept me alive. I’d have died only for them. I was too lonely for a start, and I didn’t want to go to a nursing home…’ [No needs].

## Discussion

In this study we aimed to understand more about how older recipients of home care with cognitive impairment and dementia evaluated their care-related quality of life and the extent to which self-directed homecare helps them to live well at home. This cohort with cognitive impairment rated their overall quality of life lower than other older home care users in Australia [33] but similarly to older home care users in the UK [34]. However, consistent with other research in both community dwelling older people [11] and older people, including those with cognitive impairment living in care home settings [35] this cohort perceived more favourable outcomes in relation to their life at home in lower order domains (food and drink, personal cleanliness, accommodation comfort and cleanliness and safety) than in the higher order domains (occupation and social participation). These results highlight the need to provide a greater focus within self-directed care on methods to promote outcomes within social and occupation domains for people with cognitive impairment. In the community setting, educational interventions with service providers have been used to promote improved social and occupational goal setting and engagement of older people in community settings without additional costs [36]. However, these types of interventions have not been trialled in the context of self-directed homecare and warrant further investigation.

The mixed methods approach was useful to draw out factors that older people with dementia and cognitive impairment attributed to limiting their quality of life. These included service factors, such as flexibility in the timing and the amount of support they received. Limitations resulted in some participants running out of fresh food, not being showered enough, not being able to present their homes or themselves in a way they were comfortable with, feeling lonely and having nothing to do. Overall, these had an emotional impact, contributing to a lack of control and lower self-esteem. Flexibility, reliability, and the ability to provide adequate hours of care have previously been highlighted as important in home care for older people [19]. This is especially the case for people with complex needs such as dementia, as it is often necessary to respond to changes in circumstances and reverse care decisions [37].

Limitations in personal and community mobility affected outcomes in all domains, but especially social participation and occupation. Davey [38] also found that no alternative means for private transport increased social exclusion of older people. Whilst it might be theoretically possible via consumer directed care models to negotiate more individual transport if desired, research highlights the local physical environment can itself either support or inhibit community mobility [39]. Social stigma may also affect the inclusion and participation of people with dementia. It is essential then, to understand more about how public health approaches, such as the creation of aged and dementia friendly environments are also be important to the quality of life of people with dementia living at home [40].

With regards to function within the home, participants mostly described receiving supports that substituted for lost function, either through use of technology or aides (such as walkers, handrails or personal alarms) or through the provision of direct care services (such as housecleaning or personal care). Interestingly, none in this sample described supports to regain lost function. This suggests consumer directed care may not be an effective model to promote the ‘reablement’ of older people with cognitive impairment, an approach highlighted as critical to supportive and effective dementia care [41]. Our findings are consistent with other research that suggest whilst consumer directed care may increase satisfaction with care, the approach has little effect on clinical outcomes [4].

Support to contact specialist services may be important to improving outcomes [37]. However, a self-directed care model, assumes consumers have knowledge of the value of specialist services, and also some expectation of their potential for gain (See: Swaffer for more on ‘Prescribed Disengagement™ for people with dementia [42] and also the discussion below on acceptance and adaption to disability). Australian research suggests older people have a limited understanding of the care options available to them [17] and limited resources to support their choices [43]. Further research should explore ways to promote enablement within self-directed care or whether alternative care models are more effective in this regard [41]. Consumers should be supported to make evidence informed decisions and choices to maximise their outcomes. Coaching may be a useful approach, especially when involving specialist and known health providers [44].

### Outcomes ratings vs. the lived experience

The supported interview format provided insights into the variations between participants positive QOL ratings and their sometimes less favourable qualitative responses regarding their lived experiences especially in lower order domains. This common phenomenon is often referred to as ‘response shift’ [45], reflecting shifts in internal standards or adaptation to a situation or life event. For example, Ubel et al. [46] found that when asked to rate their own health, older people implicitly compared themselves to someone their own age when the standard of comparison was unspecified, leading to higher ratings [47]. In this study some participants rated their SCRQoL higher than we might expect based on their narrative, adjusting their standards in line with what they considered acceptable for someone of their age or disability. This highlights the potential vulnerability of this older cohort and the importance of controlling for the health, functioning and intensity of care packages when interpreting survey data [35, 47, 48].

This has policy implications, particularly in the context of plans in Australia to introduce quality indicators to in-home aged care to be used by the government to measure and monitor care and the effect on service users health and wellbeing [49]. A more sophisticated indicator of care quality may be the impact services are having on SCRQoL, referred to as SCQRoL Gain [35]. This score, which can be generated using some ASCOT measures, reflects the difference services are making to people’s lives. Thus, if someone is receiving help with showering and dressing but not with social participation or occupation, we would expect to see bigger ‘gains’ in the personal cleanliness and comfort domain and no or marginal gains in the higher order domains. Those with the highest needs will have the lowest ‘expected SCRQoL’ without help and therefore have the greatest capacity to benefit from services. Thus this indicator inherently controls for differences in health and functioning, which might be impacting current quality of life, and is useful in demonstrating where there is potential to gain even when a person might have adapted to their current situation (e.g. if no difference between current and expected for a domain a service is supposed to be helping with, it would indicate a problem). There remain, however, methodological challenges around collecting this information in populations who find self-report challenging [35].

### Adaptation, acceptance and the relativity of control

Participant comments also reveal a number of important themes which help us to understand how older people with cognitive impairment think about their outcomes. Participants described adapting to living with disability through restricting their activities to create a sense of safety and control. Many also highlighted adaption to a shrinking social world. This type of accommodative coping has previously been observed in middle aged and older adults [50], people with dementia [51] and their carers [52]. However, the current findings go beyond this to highlight that accommodative coping impacts on quality-of-life ratings and could potentially obscure social care needs.

Dignity was the highest rated domain and contact with care workers was important not only for meeting practical needs, but also social and emotional needs. Continuity of care and an ability to build relationships has been previously identified in home-based care research and linked to the social dimensions as well as a sense of control for both people with dementia and their carers [53].

For this cohort, control over daily life was frequently viewed from a place of relativity. For example, the choice to ‘live in my own home’ as opposed to having to move to a care home. Many of the limitations of choice and control experienced in other domains were minimized in the context of the lived experience of staying at home. The importance of home as a place which supports maintenance of control and personal identity has been previously highlighted [54]. Our study results suggest that the overriding importance of ‘being at home’ and fear of the alternative, likely influences the way older people perceive other aspects of their quality of life. As a result, we join with colleagues in supporting the need for independent assessment of clinical and health outcomes, alongside self-reported needs or satisfaction, as part of evaluation of home care services [55].

Despite low scores on cognitive screening, only one third of participants in this study reported a diagnosis of dementia. In Australia, this has implications for the delivery of home care, as a diagnosis provides eligibility for an additional ‘dementia’ funding supplement. As such, the study also highlights the need for timely identification and diagnosis of dementia to ensure access to these additional funds. Also, funding for self-directed home care budgets for people older than 65 years is capped at a maximum budget of $59,593.55 [56]. This contrasts needs based funding for disability supports for people (< 65 years) where people with neurological conditions including Alzheimer’s Disease receive an average budget of $125, 000 [56]. In this light, government should commit to supporting cost-benefit analysis to understand the potential economic and social gains that could be experienced if older people with dementia had access to the reasonable and necessary supports they need to live well at home, rather than being forced to either adapt to disability or move into residential care. Also, given people with cognitive impairment are higher users of home care [57], governments must also be held accountable to ensure they prioritise and adopt evidence-based models of home care, such as care management, that been shown to be more effective than consumer directed care at delivering clinical outcomes for people with dementia [58].

### Limitations and future research

This was a small study in two geographical regions assessing outcomes for users of only two service providers. Whilst all users had cognitive impairment, not all had a dementia diagnosis, and it is likely their other chronic conditions also had an impact on their physical and cognitive functioning. However, the views and experiences of participants remain valid and provide us with valuable insight into the lives of older people receiving self-directed home care, as well as the difficulty associated with measuring their outcomes. Despite use of an accessible research tool and approach, the convenience sample may not reflect those with higher levels of cognitive impairment, and few were using the highest level of care package. Future research should explore alternative means of recruitment and assessment of those with dementia with higher needs who are at greatest risk of institutionalisation.

Finally, the ASCOT-ER survey administered via supported interview was useful to illustrate a clear pattern in the variance in outcomes for older people with cognitive impairment. However, preference weights used to derive SCRQoL were derived from use of the ASCOT SCT4 and are not bespoke to either the ASCOT-ER or to an Australian population. Future research may be of value to further develop the ASCOT tool for this cohort, as well as to develop preference weightings for the ASCOT-ER, and the Australian population.

## Data Availability

Data collected during this study is unable to be shared due to the conditions imposed by the ethics approval. However, study materials can be shared by contacting the corresponding author.
